# Inpatient Burden of Neurological Disorders: A Route Map for Allocation of Resources and Postgraduate Training

**DOI:** 10.7759/cureus.51311

**Published:** 2023-12-29

**Authors:** Delon Dsouza, Saji K John, Raghunandan Nadig, Shagun Bhardwaj, GRK Sarma, Sagar Badachi, Kurian Thomas, Amrutha Avati, Sonia Shivde, Thomas Mathew

**Affiliations:** 1 Neurology and Stroke, Worcestershire Acute Hospitals NHS Trust, Worcester, GBR; 2 Research, St. John's Medical College Hospital, Bengaluru, IND; 3 Stroke, St. John's Medical College Hospital, Bengaluru, IND; 4 Neuropsychology, St. John's National Academy of Health Sciences, Bengaluru, IND; 5 Neurology, St. John's Medical College Hospital, Bengaluru, IND; 6 Neurology, St. John's National Academy of Health Sciences, Bengaluru, IND

**Keywords:** inpatient neurological disorders, autoimmune disorder, demyelinating disorder, headache, epilepsy, stroke

## Abstract

Introduction

There are limited data regarding the profile of inpatient neurological disorders in India. Understanding the spectrum of diseases and the profile of patients admitted in an inpatient setting will help to streamline services, allocate resources, develop management protocols, design curricula, and improve training programs of postgraduate students in neurology training.

Objective

The objective of this study is to study the profile of inpatient neurological disorders in 1000 consecutive patients admitted to a tertiary care neurological center.

Methods

Data from 1000 consecutive inpatients admitted to the Neurology Department at St. John’s Medical College Hospital, Bengaluru from January 2018 to October 2018 were collected from the medical records. The data obtained from the case records were entered into a Microsoft Excel spreadsheet for descriptive analysis.

Results

The average age of the patients was 48 years (±18.18) and 606 of the 1000 patients were males. Strokes, including arterial and venous strokes, formed the major inpatient caseload, accounting for 48.7% of cases. Of these, 84% had ischemic arterial strokes, 7.4% had intracranial hemorrhage, and 8.4 % had cerebral sinus venous strokes; 19.3% of patients were admitted for seizures while 8.2% of patients were admitted for headache. Meningitis was diagnosed in 5.2% of patients; 4.8% of patients had central nervous system demyelinating and autoimmune diseases. A number of other diagnoses comprised less than 2.5% each and included movement disorders, peripheral nerve, spine and nerve roots disorders, neuromuscular diseases, neurodegenerative diseases, and medical and functional illness.

Conclusion

The most common disorders in the inpatient setting are stroke, seizure, headache, meningitis, and autoimmune/demyelinating disorders. These disorders should receive priority while planning the allocation of resources, educational curriculum, training, and teaching programs.

## Introduction

A systematic study of the inpatient profile of patients and their diagnosis has seldom been made in the Indian context. Many neurological disorders like migraine, benign positional vertigo, cervical and lumbosacral radiculopathies, epilepsy, and neurodegenerative diseases like Parkinson’s disease are usually managed in an outpatient setting. Inpatient admissions for neurological disorders depend on various factors like the severity of the illness, the need for time-sensitive and expedited diagnosis and treatment, associated co-morbidities, the need for intravenous medical treatment, and other interventions. Patterns of admissions also vary depending on the regional prevalence of disorders and specialization of the treating centers. This study was done to understand the spectrum of diseases for which inpatient admissions were made to a tertiary center, so that the information acquired may help to streamline services, allocate appropriate resources, develop management protocols, design curricula, and improve training programs for postgraduate students.

## Materials and methods

The study was conducted at a tertiary care neurological center in south India, with a large inpatient and outpatient burden. The neurological service at our center caters to 150 to 200 outpatients per day and 10 to 20 new inpatient admissions per day. The patients who had non-neurological diagnoses such as psychiatric disorders, systemic medical and rheumatological illnesses, and others who were transferred to units like neurosurgery were not included in this study. The final diagnosis was made after a detailed and thorough clinical evaluation by a team of four senior consultants, five junior consultants, three neuroradiologists, and six senior residents who were Doctor of Medicine (DM) trainees. All patients underwent comprehensive laboratory investigations, appropriate neuroimaging studies, and electrophysiological tests. The data obtained from the case records were entered into the Microsoft Excel database for descriptive analysis. Data entry was conducted confidentially, with exclusive access granted only to the consultants who utilized the central hospital inpatient database. Personal identifying information was deliberately masked. The data were securely stored on password-protected servers with restricted access limited to authorized personnel only. The collected data were part of ongoing registries on various neurological disorders that had prior Institutional Review Board (IRB) approvals. Descriptive statistics including mean, standard deviation, and percentages were used to summarize the data. 

## Results

The overall study duration was 10 months, from January 2018 to October 2018. This period encompassed a three-month recruitment phase during which 1000 consecutive patients were enrolled. Subsequently, data entry and analysis were conducted over the remaining seven months. There were 606 males (60.6%) and 394 (39.4%) females. The average age of the patients was 48 years (±18.18). Three-hundred and seventy five (37.5%) patients were in the 40 to 60 age group, while 272 (27.2%) patients were in the 20-40 age group and 263 (26.3%) patients were in the 60-80 age group. There were 71 (7.1%) patients below 20 years and 19 (1.9%) patients above the age of 80 years. 

Strokes including arterial and venous strokes formed the major inpatient caseload, accounting for 48.7% (487/1000) of cases. Eighty-four percent (410/487) had ischemic arterial strokes, 7.4% (36/487) had hemorrhagic strokes, and 8.4% (41/487) had venous strokes (cerebral venous sinus thrombosis); 19.3% (193/1000) of patients were admitted for seizures while 8.2 % (82/1000) of patients were admitted for headaches. Meningitis was diagnosed in 5.2% (52/1000) of patients; 4.8 % (48/1000) of patients had CNS demyelinating and autoimmune diseases. Movement disorders were seen in 2.4% (24/1000), peripheral nerve disorders in 2.2% (22/1000), spine and nerve roots disorders in 2.1% (21/1000), neuromuscular disorders in 1.8% (18/1000), cranial nerve disorders in 1.7% (17/1000), neurodegenerative disorders in 1.3% (13/1000), and medical and functional illness in 2.3% (23/1000) (Table 1). The average hospital stay was 6.9 days (Table 2).

**Table 1:** Burden of Neurological Disorders in the Inpatient Cohort (%) CVA: Cerebrovascular accident; CNS: central nervous system; CVT: cerebral venous thrombosis

Diagnosis at discharge	No of patients	%
CVA	446	44.6%
Seizures	193	19.3%
Headache	82	8.2%
Meningitis	52	5.2%
CNS demyelinating and autoimmune diseases	48	4.8%
CVT	41	4.1%
Movement Disorder	24	2.4%
Medical illness and functional disorders	23	2.3%
Peripheral Nerve disorders	22	2.2%
Spine and Nerve roots	21	2.1%
Neuromuscular disorders	18	1.8%
Cranial Nerve disorders	17	1.7%
Neurodegenerative disorders	13	1.3%
Total Number of Inpatients in the Cohort	1000	

**Table 2:** Average Duration of Hospital Stay in the Inpatient Cohort of Neurological Disorders CVA: Cerebrovascular accident; CNS: central nervous system; CVT: cerebral venous thrombosis

Diagnosis	Average No of Days
Meningitis	16.9
CVT	11.8
Neuromuscular disorders	9.1
Peripheral nerve disorders	7.8
CVA	7.7
Neurodegenerative disorders	6.1
CNS demyelinating and autoimmune diseases	5.6
Movement disorders	5.5
Spine and nerve roots	5.3
Medical illness and functional disorders	4.5
Cranial nerve disorders	4.1
Seizures	3.6
Headache	3.1
Average Duration of Hospital Stay in the Cohort	6.9

Cerebrovascular diseases

There were 487 (48.7%) patients admitted with cerebrovascular diseases. Arterial ischemic strokes were seen in 410/487(84%) of patients. Hemorrhagic venous strokes attributed to 7.4% (36/487) and 8.4% (41/487) of the stroke burden respectively.

In the 446 patients with arterial and hemorrhagic strokes, most patients had comorbidities such as hypertension which was seen in 83.4% (373/446) patients, diabetes in 53.1% (237/446) patients, and dyslipidemia in 49.1% (220/446) patients); 13.2% (59/446) of stroke patients had associated coronary artery disease. Most patients had mild to moderate strokes at admission with a median National Institutes of Health Stroke Scale (NIHSS) of 6 (Table 3) and Modified Rankin Score (MRS) of 2 or less than 2 (61.1%) (Table 4).

**Table 3:** National Institutes of Health Stroke Scale (NIHSS) Score

NIHSS score	No of patients
0-4	143
5-9	134
10-14	84
15-19	39
20-24	39
25-30	7
Grand Total	446

**Table 4:** Modified Rankin Score (MRS)

MRS	No of patients
0	17
1	161
2	94
3	61
4	91
5	21
Grand Total	445

In the 410 patients with ischemic stroke, sub-types according to Trial of ORG 10172 in acute stroke treatment (TOAST) [1]. The classifications were as follows: Large artery atherosclerosis was seen in 43.9% (180/410) patients, small vessel disease in 33.6% (138/410), cardioembolic strokes in 11.4% (47/410), strokes of undetermined etiology in 7.3% (30/410), and stroke of other determined etiology in 3.6% (15/410). Among the whole cohort of ischemic strokes, only 2.9% (12/410) were secondary to atrial fibrillation. The stroke patients spent an average of 7.7 days (± 6.6 SD) in the hospital.

Here, 8.4% (41/487) patients had cerebral venous thrombosis (CVT), and 52.5% (21/40) were males. The average age of the patients was 38.5 years (±13.14). Thirty-five patients had superficial venous system CVT while four had deep venous system CVT and two had both superficial and deep venous systems involved. Transverse and superior sagittal sinus were most commonly involved. Two patients were admitted with recurrent CVT and six patients had previous or concomitant deep vein thrombosis (DVT). On evaluation, three (7.3%) patients were found to have antiphospholipid (APLA) syndrome. This was confirmed by two consecutive tests on follow-up 12 weeks apart. Twenty-two patients (53.6%) were found to have concomitant B12 deficiency with secondary hyperhomocysteinemia and five (12.2%) patients had moderate to severe iron deficiency anemia. Two were diagnosed with CVT in the postpartum period and one had a history of oral contraceptive use.

Epilepsy

**Figure 1:**
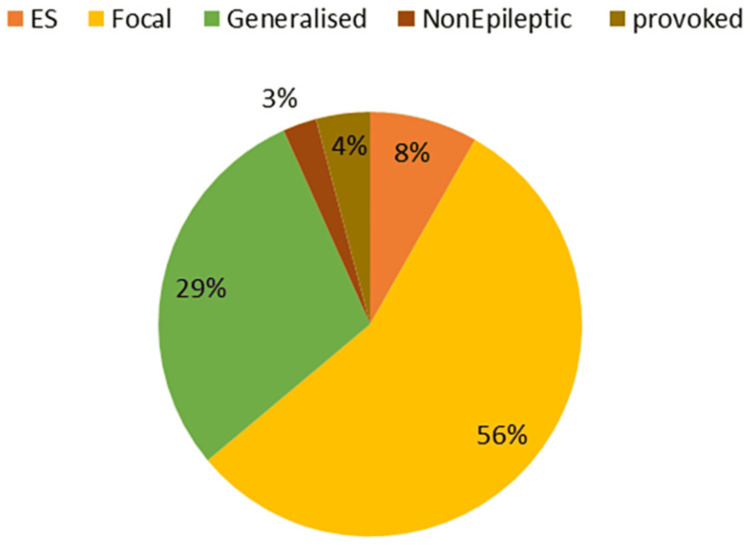
Epilepsy Types

**Figure 2:**
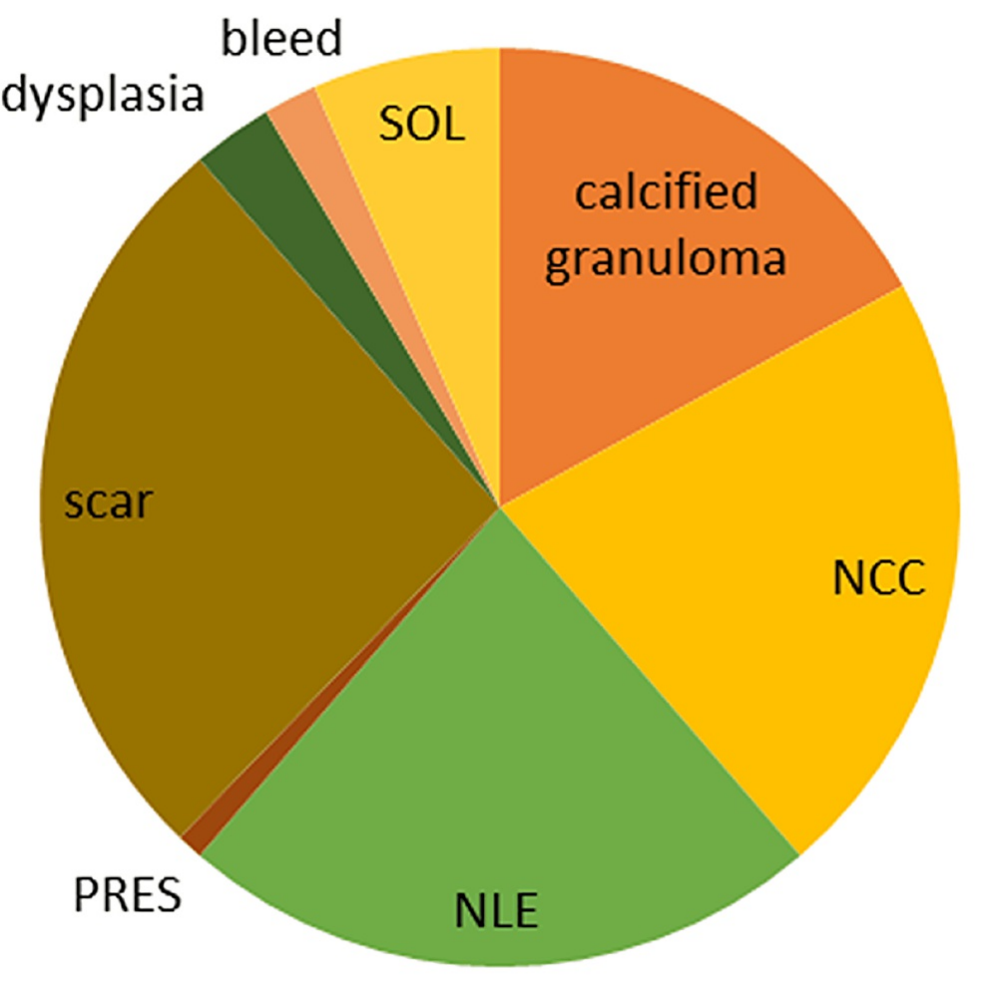
Focal Epilepsy Causes SOL: Space-occupying lesions; NCC: neurocysticercosis; NLE: nonlesional epilepsy; PRES: posterior reversible encephalopathy syndrome

19.3% (193/1000) of patients were admitted for seizures; 110 (57%) males and 83 (43%) females. The average age of the patients was 35.7 years (±18.6). 139 patients (72%) had new-onset seizures and 54 (23%) had breakthrough seizures. Five patients were admitted with status epilepticus (Figure 1). Among the 193 patients, 13 (6.7%) patients had acute symptomatic seizures due to hypoglycemia, hyperglycemia, alcohol withdrawal, and posterior reversible leukoencephalopathy syndrome (PRES). In 180 patients with unprovoked seizures, 57.7 % (104/180) had focal epilepsy, 38.3% (69/180) had generalized epilepsy, and 3.8% (7/180) patients had unknown onset epilepsy. In patients with focal onset seizures, 32.6% (34/104) were due to cortical gliosis either due to old stroke, trauma, or hypoxic injury. In 22.1 % (23/104) patients, MRI was diagnostic of neurocysticercosis and 17.3 % (18/104) of patients were found to have a calcified granuloma; 8.6% (9/104) of patients had a space-occupying lesion such as a low-grade glioma or meningioma and 1.9% (2/104) of patients were diagnosed with focal cortical dysplasia; 17.3% (18/104) patients had non-lesional focal epilepsy. Among patients with generalized onset epilepsy, five had juvenile myoclonic epilepsy, two had infantile spasms, two had Lennox-Gastaut syndrome, and one patient was diagnosed with Dravet syndrome (Figure 2).

Headache

**Figure 3:**
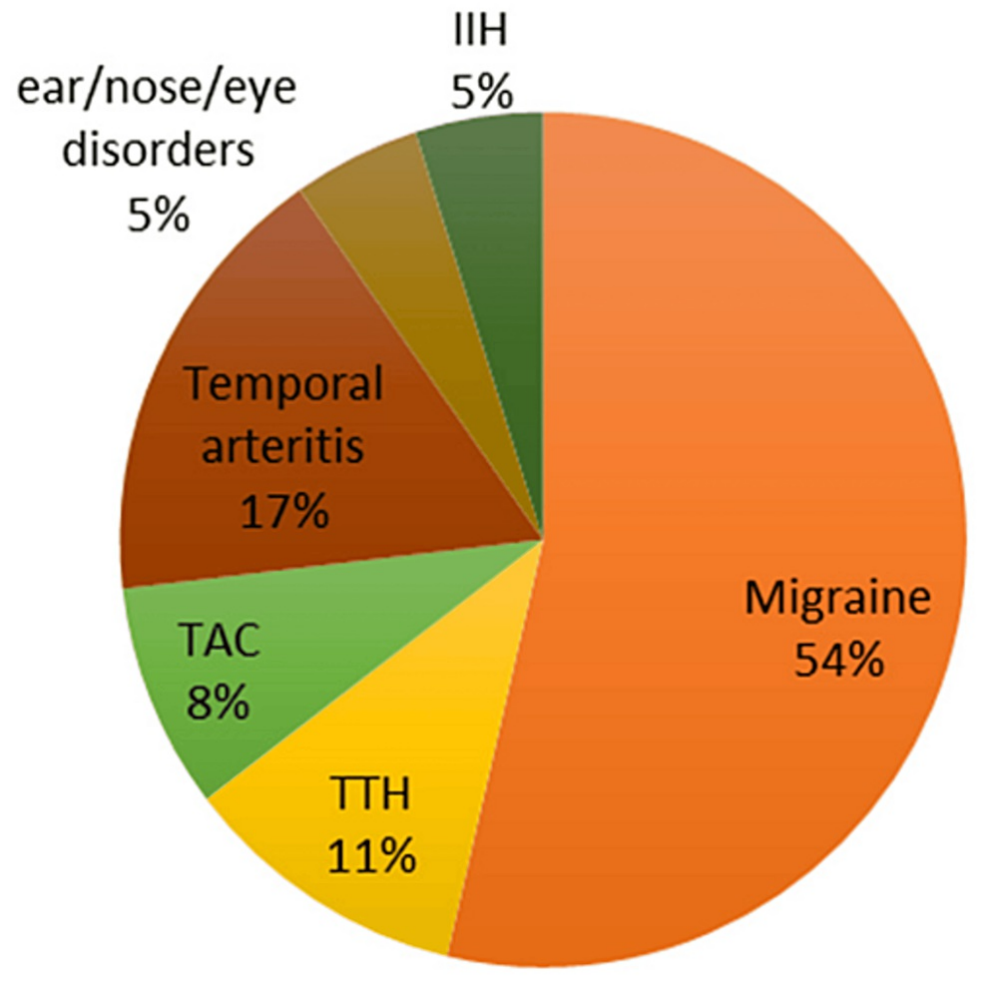
Headache Subtypes IIH: Idiopathic intracranial hypertension; TTH: tension-type headache; TAC: trigeminal autonomic cephalgia

Eighty-two (8.2%) patients were admitted for headache disorders. There were 52 (63.4%) female and 30 (36.6%) male patients. Status migrainosus was the most common reason for admission accounting for 53.7% (44/82) of the cases. The average age of the patients with migraine was 34.45 years (±14.17). Nine patients were diagnosed with tension-type headaches and seven patients had trigeminal autonomic cephalalgias of which five patients had cluster headaches, one had paroxysmal hemicrania, and one had Short-lasting, Unilateral, Neuralgiform headache attacks with Conjunctival injection, and Tearing (SUNCT) syndrome (Figure 3). Among the secondary headache disorders temporal arteritis was the most common diagnosis seen in 14 patients (17.1%), with an average age of 63.29 (±7.23) years. Other causes for secondary headaches included idiopathic intracranial hypertension, sinusitis, and glaucoma.

Meningitis

Fifty-two patients (5.2%) were admitted with meningitis. There were 28 males (53.8%) and 24 (46.1%) females. The average age of the patients was 38.2 years (±16.5). Twenty four patients (46%) had tuberculous meningitis. Twenty-two patients (42.3%) had viral meningoencephalitis of which 3 had herpes simplex meningoencephalitis. In the rest, PCR (polymerase chain reaction) was negative for the common viruses tested. Four (8%) had cryptococcal meningitis of which only one patient was human immunodeficiency virus (HIV) positive and the rest were immunocompetent patients. Only two (3.8%) patients had bacterial meningitis due to streptococcus pneumoniae.

CNS demyelinating and autoimmune disorders

Forty-eight percent (4.8%) of patients were admitted with demyelinating and autoimmune diseases. The average age of the patients was 38 years (±14.3). There were 17 (35.4%) male and 31 (64.5%) female patients. Fifty-four percent of patients (26/48) were diagnosed with multiple sclerosis, of which 21 patients had relapsing-remitting multiple sclerosis (80.7%), three patients had primary progressive multiple sclerosis (11.5%), and two (7.7%) patients had secondary progressive MS. Twenty-one percent of patients (10/48) were diagnosed with acute demyelinating encephalomyelitis (ADEM) on admission. Eight percent (4/48) had neuromyelitis optica (NMO). Eleven percent of patients (5/48) had autoimmune encephalitis, two NMDA (N-methyl-D-aspartate) antibody positive, two LG1 (leucine-rich glioma inactivated -1) antibody positive, and one GAD (anti-glutamic acid decarboxylase) antibody positive. Three patients had unclassified CNS demyelinating disorders.

Other disorders

Twenty-four patients were admitted for movement disorders, predominantly Parkinson’s disease in nine patients and multiple system atrophy in five. Patients were admitted either for a detailed evaluation or medical concurrent illness. Other movement disorders included drug-induced parkinsonism, progressive supranuclear palsy, and spinocerebellar ataxia.

 Of 22 patients with peripheral nerve disorders, six patients had Guillain Barre Syndrome and only one had chronic inflammatory demyelinating polyradiculoneuropathy. Five patients had mononeuritis multiplex of which three were diagnosed with vasculitis and two with Hansen’s neuritis on nerve biopsy. Others had diabetic plexopathy, small fiber neuropathy, and entrapment neuropathies such as wrist drops.

Of the 21 patients admitted with spine and nerve root disorders, 17 patients were diagnosed with cervical/lumbar intervertebral disc prolapse, of which 11 had only radiculopathy and six had compressive myelopathy. Other diagnoses included tuberculosis of the spine and subacute combined degeneration.

In patients with neuromuscular disorders, eight patients had myasthenia gravis (MG) and one muscle-specific tyrosine kinase (MuSK) MG. The admissions were made for impending crisis or intercurrent infections. Eight patients admitted for myopathy were diagnosed with dermatomyositis, necrotizing myositis, hypokalemic periodic paralysis, and limb-girdle muscular dystrophy.

Patients with cranial nerve disorders accounted for only 1.7% (17/1000) of admissions. Eight were admitted for refractory trigeminal neuralgia, six for Bell’s palsy, and three for post-viral 6th nerve palsy. Admitted Bell’s palsy patients were diabetics with uncontrolled glucose levels and hence admitted for blood glucose control and a short course of steroids.

2.3 % (23/1000) were admitted for medical and functional illness. Eight patients were diagnosed with dissociative disorders. Patients with neurodegenerative disorders comprised less than 1.3% of admissions. Ten patients had motor neuron disease, and two patients were admitted for dementia evaluation of which one had frontotemporal dementia and the other had Alzheimer’s dementia. One patient was diagnosed with subacute sclerosing panencephalitis.

## Discussion

The profile of neurological disorders as analyzed in 1000 consecutive inpatients in tertiary care neurological services showed cerebrovascular diseases (stroke) as the major reason for admission, contributing to 48.7% of admitted patients. Ischemic strokes were the commonest accounting for 84% of strokes. Large artery atherosclerosis was the most common stroke sub-type followed by small vessel disease. In a study from south India, in 2642 patients with stroke, over 10 years, 78.4% had ischemic strokes and 21.6% had hemorrhagic strokes. The proportion of stroke sub-types according to TOAST classification was comparable to two other large studies from India though we had a slightly larger number of small vessel diseases [2,3]. A similar distribution was seen in a study conducted at a tertiary care center in Namibia where 60.9% of the total stroke admissions had ischemic strokes and 39.1% had hemorrhagic strokes while also reporting a higher prevalence of alcohol consumption as a risk factor [4]. 

In the current study, CVT was seen in 8.4% of patients. CVT continues to be an important cause of stroke in younger patients. Studies from India have shown a 15-20% incidence of CVT in patients with stroke, especially in young strokes [5]. While earlier studies showed a preponderance of puerperal CVT, most studies done recently in India show a higher incidence of CVT in men similar to our study [5]. Anemia, both iron deficiency and B12 deficiency, with secondary hyperhomocysteinemia are important risk factors for CVT in India. The most common risk factors in women as reported by a Romanian study done over five years were pregnancy or puerperium and oral contraceptive use. The same study reported an ascending trend in the incidence of CVT with a significant increase in the age group of 18-49 [6]. 

Seizure/epilepsy was the next common reason for inpatient admission. The majority had focal seizures secondary to gliotic focus, neurocysticercosis, and calcific granuloma. In our study though, we found a higher incidence of focal onset seizures and focal epilepsy, most epidemiological studies in India and other developing countries have shown a greater proportion of generalized seizures and generalized epilepsies [7]. A study from eastern China by Yu et al. (2019) reported cardiovascular diseases as the primary cause of hospitalization in patients with epilepsy [8]. In our study, however, none of the patients with epilepsy were admitted for cardiovascular diseases. In high-income countries, a greater proportion of patients had focal epilepsies [9]. The higher proportion of focal epilepsies in our study is probably due to the use of MRI, prolonged EEG monitoring, and the use of the new International League Against Epilepsy (ILAE) 2017 classification for seizures and epilepsy when compared to most other studies from India, which used only a CT scan for diagnosis. 

Headaches were the third reason for admission and the majority of the admissions were for the treatment of status migrainosus. This is an interesting observation and shows the severity and burden of headache disorders. Though headache is treated on an outpatient basis and is one of the most common diagnoses in OPD neurology clinics, [10] migraine continues to be an important reason for inpatient admissions. Studies from Western countries like the USA have shown, 63% of admitted headaches were for treatment of migraine [11]. Similarly, in a district general hospital in England, 17.7% of neurology admissions were for headaches [12]. In India though, there are several studies of headache burden in the community and outpatient setting, a systematic study on the inpatient burden of headache is not found.

Meningitis was the fourth most common reason for inpatient admission. The most common type of meningitis in the inpatient setting was tuberculous meningitis, followed by viral meningitis. In a study done in India on the clinical profile of 984 meningitis patients admitted to a tertiary care center, tuberculous meningitis (55%) was the most common followed by viral (22.7%) and then bacterial meningitis (13.9%) [13]. This shows that tuberculous meningitis is still of utmost priority in our country.

Demyelinating and autoimmune disorders also contributed to the inpatient load, with relapsing-remitting multiple sclerosis being the commonest. There are few studies concerning the prevalence and spectrum of demyelinating disorders in India. In a study from South India, of 79 patients with demyelinating diseases, 35 had multiple sclerosis. Twenty-six patients had clinically isolated syndrome, 11 had NMO and NMO spectrum disorders, and six had ADEM [14]. We saw a similar distribution of demyelinating disorders in our inpatients for demyelinating disorders.

According to our data, stroke medicine and stroke care should receive the top priority among all neurological disorders, followed by seizures/epilepsy, headache, meningitis, autoimmune and demyelinating disorders. As the priority of any teaching curriculum is to enable residents to manage common neurological disorders in the community, the teaching curriculum and educational activities should give more thrust to these disorders. In the area of teaching and examination, it is recommended that the curriculum should follow a pattern of “must know” subjects, which should constitute 70%, “important to know subjects” constituting 20%, and “good to know” subjects constituting 10%. The prevalence of neurological diseases in comparison to other conditions is relatively larger as reported by large-scale global studies and therefore requires a greater emphasis in teaching curricula in low- and middle-income countries [15].

Based on our observations, we suggest including stroke, epilepsy, headache, and meningitis in the category of “must know” and other disorders like autoimmune and demyelinating neurological disorders in the “important to know” category. More time and resources should be allotted for education, training, and management of these “must-know” disorders. These resources may include didactic lectures, case presentations, group discussions, journal clubs, workshops, and seminars. This may enable the postgraduates to learn and manage common neurological ailments when they complete their training course. Even benchmarks for passing examinations can be based on the knowledge of these “common disorders. Standard protocols should be made for the treatment of these common disorders across the centers so that management will be uniform across the country.

There are a few important limitations of the study. It was a single-center study and it may not represent the pattern of admissions at the various centers across the country. Neurodegenerative disorders formed a very small proportion of cases in our study. The presence of other large tertiary care centers both governmental and private, in the neighborhood may be one of the causes of these discrepancies. Also, common neurodegenerative diseases like Alzheimer's disease and Parkinson's disorder are usually managed in the outpatient setting. Newly described disorders like myelin oligodendrocyte glycoprotein (MOG)-associated disease would have been missed as the data were collected in 2018. Though we have accounted for all patients admitted in the neurology department, a few patients admitted under medicine or pediatrics with neurological disorders might have been unaccounted for. Due to referral bias, there may be an overrepresentation of multiple sclerosis patients at our center. This may be true for other institutes that have specialized care for disorders like epilepsy, movement disorders, ataxia, dementia, etc. where there may be an over-representation of these disorders due to referral bias. Future studies of a similar nature involving a larger number of people from different centers across various regions of the country may give us a more comprehensive picture of the inpatient neurological disorder burden in the country.

## Conclusions

The most common five neurological disorders for which patients were admitted were stroke, seizure, headache, meningitis, and autoimmune/demyelinating disorders. These five disorders should be given priority in resource allocation, teaching curricula, training programs, and neurology examinations. This will enable us to have standardized protocols and streamline neurological care across the country. This will also empower qualifying postgraduates to manage the common neurological disorders prevalent in the population confidently and effectively.
